# Evaluation of immune responses following infection of ponies with an EHV-1 ORF1/2 deletion mutant

**DOI:** 10.1186/1297-9716-42-23

**Published:** 2011-02-07

**Authors:** Gisela Soboll Hussey, Stephen B Hussey, Bettina Wagner, David W Horohov, Gerlinde R Van de Walle, Nikolaus Osterrieder, Lutz S Goehring, Sangeeta Rao, David P Lunn

**Affiliations:** 1 Department of Clinical Sciences, College of Veterinary Medicine and Biomedical Sciences, Colorado State University, 300 W. Drake Rd, Fort Collins, Colorado 80523, USA; 2 Department of Population Medicine and Diagnostic Sciences, College of Veterinary Medicine, Cornell University, Ithaca, New York 1485, USA; 3 Department of Veterinary Sciences, University of Kentucky, Lexington, KY 40546, USA; 4 Department of Comparative Physiology and Biometrics, Faculty of Veterinary Medicine, Ghent University, Salisburylaan 133, 9820 Merelbeke, Belgium; 5 Institut für Virologie, Freie Universität Berlin, 10115 Berlin, Germany

## Abstract

Equine herpesvirus-1 (EHV-1) infection remains a significant problem despite the widespread use of vaccines. The inability to generate a protective immune response to EHV-1 vaccination or infection is thought to be due to immunomodulatory properties of the virus, and the ORF1 and ORF2 gene products have been hypothesized as potential candidates with immunoregulatory properties. A pony infection study was performed to define immune responses to EHV-1, and to determine if an EHV-1 ORF1/2 deletion mutant (ΔORF1/2) would have different disease and immunoregulatory effects compared to wild type EHV-1 (WT). Infection with either virus led to cytokine responses that coincided with the course of clinical disease, particularly the biphasic pyrexia, which correlates with respiratory disease and viremia, respectively. Similarly, both viruses caused suppression of proliferative T-cell responses on day 7 post infection (pi). The ΔORF1/ORF2 virus caused significantly shorter primary pyrexia and significantly reduced nasal shedding, and an attenuated decrease in PBMC IL-8 as well as increased Tbet responses compared to WT-infected ponies. In conclusion, our findings are (i) that infection of ponies with EHV-1 leads to modulation of immune responses, which are correlated with disease pathogenesis, and (ii) that the ORF1/2 genes are of importance for disease outcome and modulation of cytokine responses.

## Introduction

Equine herpesvirus-1 remains one of the most common viral infections of horses causing respiratory disease, epidemic abortion, and outbreaks of equine herpes myeloencephalopathy (EHM) [1]. Primary infections with EHV-1 lead to establishment of latent infection within the first weeks or months of life. The two main strategies for controlling EHV-1 infection and disease are management practices and vaccination, however immunity established after either infection or vaccination is short lived and incomplete [1].

Equine adaptive immune responses and protection from EHV-1 have been extensively studied. While virus-neutralizing (VN) antibodies play a role in reduction of nasal viral shedding [2], cytotoxic T-lymphocytes (CTLs) are most critical for protection from clinical disease, viremia and nasal viral shedding [2-4]. In contrast, innate immunity to EHV-1 infection is poorly characterized. Innate immunity in mice and humans has been demonstrated to be critically important for immediate protection as well as for shaping subsequent adaptive immune responses via initial interaction of viral pathogens with pattern recognition receptors (PRR) that prime and direct subsequent immunological events [5]. Characterization of early and innate responses to EHV-1 may help explain the hosts failure to generate long-lasting immunity.

Viruses have developed an array of strategies to circumvent host immunity, and for EHV-1 it is thought that the lack of long-lasting immunity is due to immunomodulatory properties of the virus [6-11]. Strategies employed by EHV-1 include interference and modulation of NK-cell lysis, alteration of cytokine network responses that ultimately affect B- and T-cell responses, loss of efficient antigen presentation and chemoattraction of professional antigen presenting cells, antibody dependent cytotoxicity, and CTL responses [12].

Most research on EHV-1 immunomodulation has been performed in vitro or using mouse models. Few in vivo equine studies have been performed [8,13,14] and these have focused on clinical outcomes and viremia while innate and early immune responses were not examined in detail. All EHV-1 genes are expressed within the first hours of infection, and may therefore target early innate immune responses long before the onset of an adaptive immune response.

**Figure 1:**
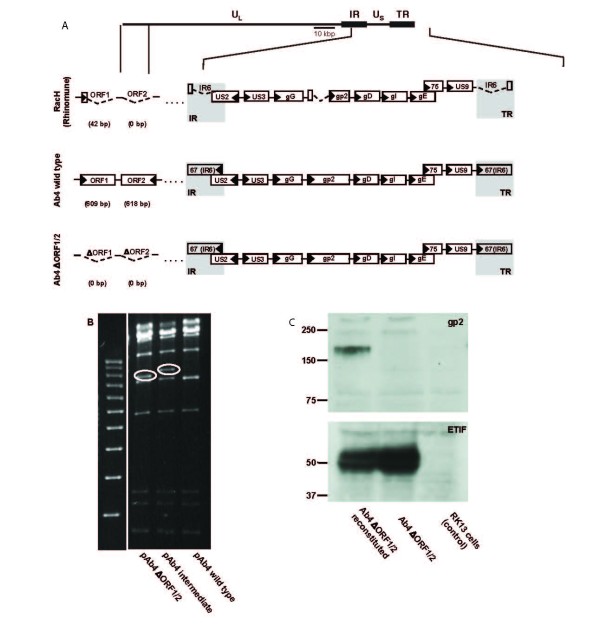
**(A). Genomic organization of RacH, Ab4 wild type and the recombinant Ab4 OFR1/2 deletion mutant**. Shown is the RacH and Ab4genome with a detailed organization of parts of the unique long (UL) and unique short (US) regions, along with parts of the inverted and terminal repeat regions (IR & TR, shaded in grey). In addition, the genome of the recombinant Ab4 ORF1/2 mutant is shown where the genes ORF1 and ORF2 were deleted. **B**. Restriction fragment length polymorphisms (RFLPs) that confirm correct deletion of the ORF1 and 2 genes in the pAb4 background. BAC DNA was digested with NotI and separated by electrophoresis on a 0.8% agarose gel. Staining of the gel with ethidium bromide (EtBr) shows changes in RFLP patters obtained, indicated by circles, after the first recombination (insertion of the aphA1 gene; pAb4 intermediate) and the second recombination (removal of aphA1 gene and the ORF1/2 genes; pAb4ΔORF1/2). **(C)**. Western blot analysis detecting the expression of gp2 in cellysates of cells infected with reconstituted or not reconstituted Ab4ΔORF1/2. Mock-infected cells (RK13 cells) were included as controls. Cellysates were loaded and separated by SDS-12% PAGE under reducing conditions. Separated proteins were transferred to a nitrocellulose membrane and incubated with monoclonal antibodies (mAb) 3B12 (against gp2; 1:10) or the control mAb L3ab (against ETIF, 1:2000). The sizes of the PageRuler Prestained protein Ladder (Fermentas) are given in thousands.

Amongst current EHV-1 vaccines in use, modified live vaccines (MLV) typically perform best [15]. Studies have shown clinical and virological protection from EHV-1 infection after MLV vaccination with attenuated EHV-1 strains (RacH, NY03-H3) containing deletions in the IR6 gene and the left terminus of the genome (ORF1/2 genes) [16-18] (Figure 1a). The IR6 gene has already been intensively studied in vitro as well as in vivo [19-21], but no information is available to date regarding the functions of the ORF1/2 genes. Based on the fact that the genes ORF1 and 2 are (i) expressed very early in infection and (ii) absent in the attenuated RacH strain, we choose to study their possible immunoregulatory role in an equine model. For this purpose, a recombinant Ab4 mutant was generated where the ORF1 and ORF2 genes were deleted (Ab4ΔORF1/2) (Figure 1a). Ponies were infected with Ab4 wild type (WT) or ΔORF1/2 virus and the effects on innate and adaptive immune responses, and on severity of clinical disease, nasal viral shedding and viremia was determined.

## Material and methods

### Experimental animals

Nineteen yearling ponies of both sexes were used for this study. All ponies were tested for prior exposure to EHV-1 using virus neutralization tests and none of the ponies exhibited titers > 128. Ponies were fed twice a day with a diet of hay and pelleted concentrate. The maintenance and experimental protocols followed the animal care guidelines of the Animal Care and Use Committee, Colorado State University.

### Equine herpesvirus virus preparation, isolation and kinetics

This experiment used the neuropathogenic EHV-1 strain Ab4, a native equine strain that is known to cause respiratory disease, abortions and neurological disease. In addition, an Ab4 mutant virus lacking the ORF1 and ORF2 genes (Ab4ΔORF1/2) was generated as follows. A recombinant BAC, pAb4ΔORF1/2 was generated by two-step Red-mediated (*en passent*) recombination [19] using the mutagenesis primers ORF1/2_FW: 5'CGAGGGAGTTTCGCGGGGCCGCGCCTCCTCTGTCTCCAT CGGTAGGGTTTGAGACGTGTAAGGATGACGCGATAAGTAGGG3' and ORF1/2_RV:5'TCCAGCGTGGAGGAGGCGCATACACGTCTCAAACCCTACCGATGGAGACA GAGGAGGCGCCAACCAATTAACC AATTCTGATTAG 3' (underlined sequence indicates the template binding region of the primers for PCR amplification with pEPKanS). Parental and mutant BAC DNA was isolated, digested with *Not*I and analyzed by 0.8% agarose gel electrophoresis after visualization of DNA fragments with ethidium bromide (EtBr) (Figure 1b). In addition, the identity of the mutant BAC DNA's was also confirmed by sequencing, using the primers ORF1/2-F (5' CCCTCT ACGGTTTTCTTCGAGGCCG 3') and ORF1/2-R (5' CCTAGGCGATG TGTGCAGCCGAGGC 3') (data not shown). Virus was reconstituted after co-transfection of 5 μg of BAC DNA into rabbit kidney (RK13) cells with 2 μg of plasmid DNA expressing the full-length gp2, using the CaPO_4 _precipitation method as previously described [22,23]. At day 5 after co-transfection, supernatants were harvested and transferred to fresh plates of RK13 cells, and recombinant non fluorescing virus plaques were picked and purified to homogeneity by two rounds of plaque purification [22]. To detect gp2 expression, the monoclonal antibody (MAb) 3B12 was used and western blotting was performed exactly as described previously [23,24] (Figure 1c). Before in vivo inoculation of ponies with these strains, the growth kinetics of Ab4 WT and Ab4 ΔORF1/2 were compared in RK13 cells and equine dermal cells (NBL6) grown in minimal essential medium (Sigma-Aldrich, St. Louis, MO, USA) supplemented with 10% fetal bovine serum (FBS), 100 U/mL penicillin and 0.1 mg/mL streptomycin (Sigma-Aldrich) in three independent experiments. For this purpose, 1 × 10^5 ^RK13 or NBL-6 cells were infected at a multiplicity of infection (MOI) of 3. Virus was allowed to attach to the cells for 30 min at 4°C, followed by a 1.5 h penetration period at 37°C, and then all excess virus was washed off. At different time points post infection (pi), cells and supernatants were collected and intra- and extracellular viral titers were determined by plating onto RK13 cells.

### Experimental design and infection

Nineteen ponies were randomly assigned to 3 groups. Group 1 (*n *= 7) were infected with Ab4 WT virus (Ab4 WT group), group 2 (*n *= 7) were infected with Ab4 ΔORF1/2 (Ab4 ΔORF1/2 group) and group 3 (*n *= 5) were uninfected ponies (Controls). The infection groups were infected by intranasal instillation of 1 × 10^7 ^PFU of the respective viruses in 7mLs of saline. During the study ponies were housed in geographically separate groups, and kept separate from all other horses. Infections and sample collections were performed by separate teams so as to avoid cross contamination of the different experimental groups.

### Clinical data

Physical examinations were conducted prior to infection and daily for thirteen days and every other day from day 14 to day 21 pi. For each day, a total clinical score was calculated by checking for the presence of five clinical signs (cough, fever defined as a rectal temperature > 38.6°C, ocular discharge, nasal discharge and depression) as described previously [25]. Each of these clinical signs received a score of 1 if present and 0 if absent in order to calculate the total clinical score per animal.

### Sample collection

Experimental samples were taken as indicated in Table 1. Nasal swab samples for virus isolation were collected using Dacron swabs (Baxter Healthcare Corporation, McGaw Park, IL, USA) and were stored in 1 mL of virus transport medium (PBS containing 5% glycerol, 800 U m/L Penicillin/Streptomycin, 200 U m/L Gentamycin, and 100 U m/L Nystatin) at -70°C. Blood for detection of cell-associated viremia was collected by jugular venipuncture into heparinized tubes. Blood for detection of neutralizing antibodies was collected by jugular venipuncture into serum separator tubes (BD, Franklin Lake, NJ, USA) and serum samples were aliquoted and stored at -20°C until analysis. Blood for cytokine measurement was collected by jugular venipuncture using the PAXgene™ RNA system (BD) and RNA was isolated and processed according to manufacturer's instructions. Nasal secretions for cytokine measurement were collected using tampons in the ventral nasal meatus for a minimum of 20 min as previously described [3], and stored at -20°C until analysis. For lymphoproliferative responses and cytokine production following re-stimulation, blood was collected into heparin as described previously [25].

**Table 1:** Experimental design

Day of study*	- 2	0	1	2	3	4	5	6	7		9		14		21	56	78	96
Physical Exam	X		X	X	X	X	X	X	X	X	X	X	X	ad	X			
Shedding &Virema	X		X	X	X	X	X	X	X	X	X	X	X	ad	X			
SN titers	X			X					X				X		X	X	X	
Nasal cytokines	X		X	X				X										
Paxgene mRNA	X		X	X	X	X	X	X	X									
Re-call responses:																		
-Prolif.	X						X		X						X	X	X	X
-Cytokines																X	X	X

*Day 0: day of infection; ^+^ad indicates sampling on alternating days

### Nasal virus shedding

Viral DNA in nasal swab samples was isolated using the Qiamp DNA blood minikit, according to manufacturer's instructions (Qiagen, Inc., Valencia, CA, USA). To determine nasal virus shedding, viral DNA load was determined by real time PCR using a specific probe recognizing glycoprotein B (gB) of EHV-1, as previously described [26]. To ensure no cross-contamination had occurred between the different experimental groups, nasal swab DNA on day 2 pi from all experimental animals was tested for presence of Ab4 WT or Ab4 ΔORF1/2 using the following primer set, based on the published Ab4 sequence (Acc # N_001491): FP: 5'- CAACAACCCTGGGCTCTTTA -3'(5' at position 80 flanking the ORF1gene) and RP: 5'- GATTCGCACCTCATCTCCAC -3'(5' at position 2132 flanking the ORF 2 gene), at standard cycling conditions. Using these primers, the expected product for ponies infected with Ab4 WT would be 1.25 kb and around 0.4 kb for ponies infected with Ab4 ΔORF1/2. The PCR results confirmed that no cross-contamination with the different viruses between the pony groups had occurred (data not shown).

### Cell-associated viremia

For detection of cell-associated viremia, peripheral blood mononuclear cells (PBMCs) were isolated from heparinized blood using density gradient centrifugation over Histopaque-1077 (Sigma, St. Louis, MO, USA). Viral and cellular DNA was isolated using the Qiamp DNA blood minikit according to manufacturer's instructions (Qiagen, Inc.). Viral load was determined by real time PCR, as described for determining viral load in nasal swabs in the above section. Beta actin was used as the cellular housekeeping gene using primer and probe sequences developed previously [27]. Viral load was expressed as the log of EHV-1 gB DNA copies/10^6 ^β-actin copies.

### Antibody responses to EHV-1

Virus neutralizing antibody titers in serum sample were determined as described previously [28]. Antibody responses were also independently evaluated using a kinetic enzyme-linked immune absorbent assay (ELISA) as described previously [18]. This kinetic ELISA was used because it typically allows for more accurate differentiation between the IgG isotype responses when compared to a conventional endpoint ELISA due to decreased variability and measurement as a continuous titer. Briefly, microtiter plates (MaxiSorb, Nalge Nunc Int., Rochester, NY, USA) were coated with EHV-1 virus antigen in a final concentration of 5 μg/mL. After washing, serum samples and EHV-1 positive and negative control sera were diluted 1:200 in phosphate buffer and incubated on plates followed by washing and addition of cell culture supernatants of monoclonal antibodies (Mab) recognizing each of the equine IgG sub-isotypes (IgGa, IgGb, IgGc and IgG(T)). A peroxidase conjugated goat anti-mouse IgG(H + L) antibody (Jackson ImmunoResearch, West Grove, PA, USA) was added before washing and addition of substrate solution. Kinetic plate reads were obtained at 45, 90 and 135 seconds after substrate addition in an ELISA reader (ELx808, Biotek Instruments Inc., Winooski, VT, USA) at 630 nm and the generated slope value for each sample was multiplied by 1 000 for data presentation.

### Measurement of cytokine mRNA expression in blood

Total RNA from PAXgene™ tubes was extracted according to manufacturers instructions and 1 μg of RNA was reverse-transcribed. Cytokine/chemokine specific cDNA was quantified by real-time PCR using equine-specific, intron-spanning primers for IL-1, IL-2, IL-8, IL-10, IL-12, IFN-γ, TNF-α, TGF-β, FoxP3, Tbet, GATA3 and the housekeeping gene β-gus with an Applied Biosystems 7900 sequence detection system. For primer and probe sequences see [29]. Relative quantity (RQ) of each gene was determined with the 2(-Delta Delta C(T)) method [30] using the average day -2 values of all horses for each gene as calibrators.

### Measurement of IL-4, IL-10, IFN-α in nasal secretions

Nasal secretions were tested for the presence of IL-4, IL-10 and IFN-α with a fluorescent bead-based system (Luminex IS 100 instrument, Luminex Corp. Austin, TX, USA) [31] as previously described [32]. The data were reported as median fluorescent intensities. For standard curve fitting and subsequent calculation of concentrations in samples the logistic 5*p *formula (*y *= *a *+ *b*/(1 + (*x*/*c*)^*d*)^*f*) was used (Luminex 100 Integrated System 2.3).

### Non-specific and EHV-1-specific lymphoproliferative responses

For measurement of lymphoproliferative responses in lymphocyte subsets, 5-6-Carboxyfluorescein diacetate succinimidyl ester (CFSE) was used to stain and detect cell division using the CyAn™ ADP - Multiparameter Flow Cytometer (Dako Corporation, Fort Collins, CO, USA). Isolated PBMCs were re-suspended at 1 × 10^7 ^in 1 mL of PBS before adding CFSE at a concentration of 5 μM. After 8 min incubation at room temperature, the reaction was quenched using 2 mL FCS and washed twice. Cells were re-suspended in media (RPMI 1640 containing 2 mM L-glutamine, streptomycin 10 mg/mL, penicillin 10 000 units/mL, 2 mM sodium pyruvate, 2 mM β-mercaptoethanol, 10 mM HEPES and10% FCS; Gibco/Invitrogen, Carlsbad, CA, USA). Stained and unstained cells were plated in 24 well tissue culture plates (Corning Inc., NY, USA) at 2 × 10^6 ^cells/well before adding media, heat-inactivated Ab4 at an MOI of 5, or PHA at 0.2 or 1 μg/mL (Sigma, St. Louis, MO, USA) incubation in a humidified 37°C incubator with 5% CO_2_. Following a 5-day incubation period, cells were harvested by centrifugation at 300 *g *and re-suspended in FACS buffer (PBS with 0.4% FCS, and 0.1% NaNH_3_) before plating and staining aliquots with cell supernatants of the following anti-equine monoclonal antibodies: CVS19 (anti-equine CD13 control Ab), CVS4 (anti-equine CD4), CV8 (anti-equine CD8) [33]. After washing, cells were stained with Goat anti-mouse IgG-1 Tricolor conjugate (Caltag Laboratories, Burlingame, CA, USA) for detection. B cells were stained using a directly conjugated goat α-equine IgG light chain antibody (Jackson ImmunoResearch, West Grove, PA, USA) at a 1:10 dilution. Lymphoproliferative responses for each cell subset were analyzed using flow cytometry and calculated by subtracting the percent of proliferating cells stimulated with media alone from the percent of proliferating cells stimulated with either EHV-1 or PHA.

### Cytokine mRNA responses to in vitro re-stimulation with EHV-1

IFN-γ, Granzyme B, and Perforin mRNA responses to re-stimulation with EHV-1 was examined by real time PCR as described above. For re-stimulation experiments, PBMCs collected at days 56, 74 and 96 pi were plated at a concentration of 4 × 10^6 ^cells in 1.2 mL of media per well, and re-stimulated with either heat-inactivated EHV-1/Ab4 at a MOI of 5 or incubated with media alone or 1 μg mL of PHA (Sigma) in a humidified 37°C, 5% CO_2 _incubator for 48 h. Cells were collected by pelleting at 300 g for 3 min. Total RNA was recovered using TRIzol and the RNeasy Mini Kit protocol plus DNAse treatment (Quiagen, Inc.) and reverse transcribed. IFN-γ, Granzyme B, and Perforin cDNA was measured by real time PCR assay using specific probes and primers as described above.

### Statistical analysis

All continuous data were evaluated for assumptions of normality prior to performing a linear regression analysis. The overall effect of both virus groups on the outcome, when compared to uninfected control group, was determined using a multivariable linear regression analysis. The difference between the experimental groups on each day pi was evaluated using a bivariable linear regression analysis stratified by "day". A separate analysis to compare post-infection days to the pre-infection day was performed within each virus group. The data on temperature and viremia was dichotomized to evaluate the probability of occurrence of fever or viremia in the two infection groups when compared to the controls, using a multivariable logistic regression analysis taking into account the repeated measurements on ponies across different days. A bivariable logistic regression analysis was performed to evaluate the probability of occurrence of fever on febrile days and viremia on viremic days in the Ab4 ΔORF1/2-infected group compared to the WT-infected group. When multiple comparisons occurred within one analysis, a Bonferroni adjustment of *p*-value was used to evaluate statistical significance. A *p*-value of < 0.05 was used to determine statistical significance.

## Results

### Growth kinetics of Ab4 WT and Ab4 ΔORF1/2

Growth kinetics of Ab4 WT and Ab4 ΔORF1/2 were evaluated in RK13, (Additional file 1: Figure S1) and NBL6 cells (not shown). Measurement of both intracellular and extracellular virus titers showed that deletion of the ORF1/ORF2 genes did not alter in vitro growth kinetics when compared to Ab4 WT.

### Clinical signs following EHV-1 infection

**Figure 2:**
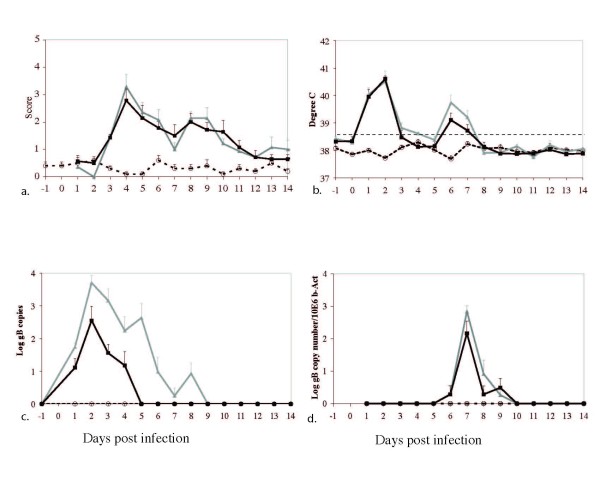
**Mean clinical score, body temperature, viral nasal shedding, and viremia following infection**. Clinical scores (a). Body temperatures are indicated in degree Celcius and the dotted line indicates fever (defined as a T ≥ 38.6°C) (b). Viral nasal shedding is represented as mean log gB copy numbers in nasal swabs as determined by real time PCR (c) and viremia is represented as log gB copy numbers/106 copies of β-actin in PBMCs (d). Controls (*n *= 5): white circles, Ab4 WT infected (*n *= 7): grey triangles, Ab4 delta ORF1/2 infected (*n *= 7): black squares. Data are displayed as means + SEM.

Animals in both infection groups showed signs of EHV-1 respiratory disease including pyrexia, lethargy, ocular and nasal discharge, and coughing between day 1 and day 14 pi, while none of the uninfected control animals showed any sign of clinical disease (Figure 2a). Differences in clinical scores were not significantly different between Ab4 WT- and Ab4 ΔORF1/2-infected ponies. Body temperatures are shown in Figure 2b and temperatures of both infection groups were significantly higher then in uninfected controls (*p *< 0.0001). The duration of the primary fever was significantly shorter in Ab4 ΔORF1/2-infected ponies compared to Ab4 WT-infected ponies (*p *< 0.0001).

### Nasal virus shedding and cell-associated viremia

Nasal virus isolation and viremia results are shown in Figure 1c and 1d respectively. As observed for clinical data, only animals in infection groups exhibited nasal viral shedding and viremia, while no viral DNA was detected from uninfected controls throughout the experiment. Infection with Ab4 ΔORF1/2 resulted in reduced nasal virus shedding when compared to Ab4 WT, both in terms of duration and magnitude of virus shedding (Figure 2c), and these differences were statistically significant from days 1 through 5 post-infection, (*p *= 0.0005). Viremia results are shown in Figure 2d; both infection groups were viremic over multiple days, while control animals were not viremic (*p *< 0.001). Viremia was not significantly different between the infected groups.

### Antibody responses

**Figure 3:**
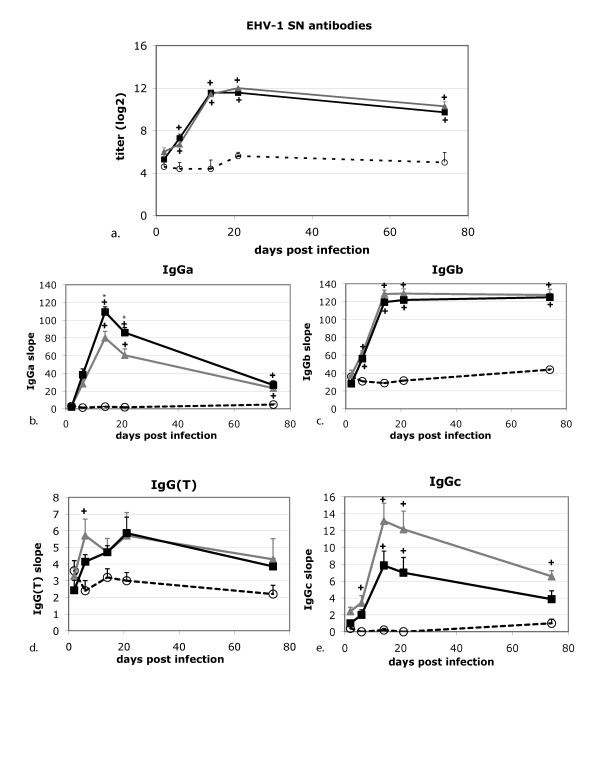
**EHV-1 serum neutralization titers (a) and kinetic ELISA IgG subclass anti-EHV-1 antibody responses (b-e)**. Controls (*n *= 5): white circles, Ab4 WT infected (*n *= 7): grey triangles, Ab4 delta ORF1/2 infected (*n *= 7): black squares. Data are displayed as means + SEM. Asterisks indicate statistically significant differences between infection groups, + indicates significant differences with the controls (*p *< 0.05).

Serum neutralization (SN) titers and antibody isotype responses are shown in Figure 3. SN titer responses are displayed after log transformation, because SN titers were not normally distributed. No increases in antibody responses occurred prior to infection. Following infection, SN titers, IgGa, IgGb and IgGc antibodies in both infection groups increased significantly when compared to the controls (*p*-values: SN titers < 0.001, IgGa < 0.04, IgGb < 0.04, IgGc < 0.04). SN titers started to rise by day 6 pi in both infection groups, peaked by day 21 and slowly declined but were still well above pre-infection levels on day 74 pi (Figure 3a). SN titers were not significantly different between the infection groups. IgGa antibody responses to EHV-1 were apparent following infection with both viruses. Responses in the Ab4 ΔORF1/2-infected group were significantly higher on days 14 and 21 pi as compared to the Ab4 WT-infected group (*p *= 0.01 and 0.03 respectively, Figure 3b). Overall, the pattern of IgGa responses were of a shorter duration compared to SN responses. In contrast, the IgGb responses paralleled the pattern of SN responses in both infection groups, and no significant differences between infection groups were observed (Figure 3c). The IgG(T) responses differed from all other antibody response patterns and were of very low amplitude (Figure 3d). Pre-existing anti-EHV-1 IgG(T) antibody responses were detectable in all groups and increased upon infection, but increases in anti-EHV-1 IgG(T) levels were only statistically significant in the Ab4 ΔORF1/2-infected group (day 14 pi *p *= 0.007, day 21 pi *p *< 0.0001). The IgGc responses were of low amplitude, and did not differ between infection groups (Figure 3e).

### Cytokine expression in blood and nasal secretions

**Figure 4:**
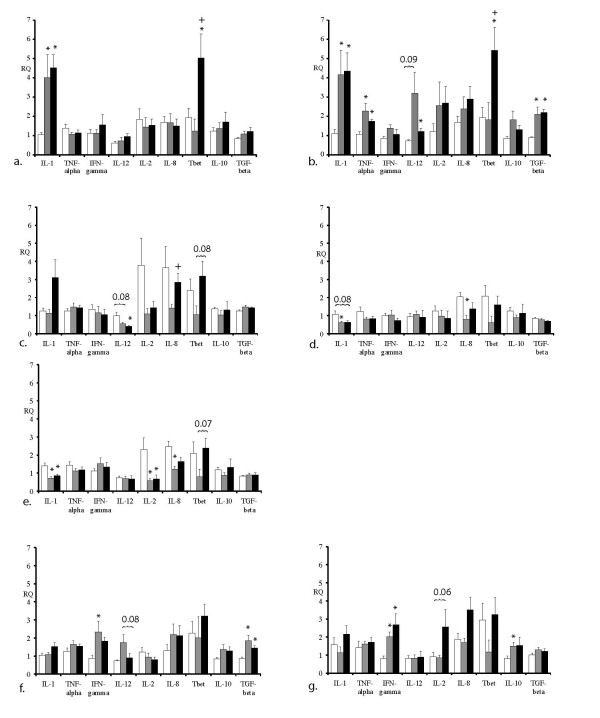
**EHV-1 specific cytokine mRNA responses at days 1-7 pi**. Relative quantities (RQ) represent x-fold increases over average levels observed for the same cytokine on day -2 prior the experimental infections. Controls (*n *= 5): white bars, Ab4 WT infected (*n *= 7): grey bars, Ab4 delta ORF1/2 infected (*n *= 7): black bars. Days 1-7 pi are represented by figure a-g respectively. Data are displayed as means + SEM. A statistical difference from the control group is indicated by an * (*p *< 0.05) and between the two infection groups by an + (*p *< 0.05).

Figure 4 shows the relative quantity (RQ) values of cytokine expression levels in blood samples at days 1-7 pi. The major findings were that (i) Infection with both viruses resulted in increased cytokine responses on days 1 and/or 2 pi (IL-1, TNF-α, and TGF-β in Ab4 WT-infection group; IL-1, TNF-α, IL-12, Tbet, and TGF-β in Ab4 delta ORF1/2 infection group) and days 6 and/or 7 pi, (INF-γ, and TGF-β in both infection groups, IL-10 significant in the Ab4 delta ORF1/2 infection group only) coinciding with the biphasic temperature response and onset of respiratory disease and viremia, while cytokine mRNA expression (IL-1, IL-2, IL-8) was decreased in the Ab4 WT infection group when compared to uninfected controls on days 3, 4, and 5 pi, (ii) Tbet mRNA expression in PBMCs of the Ab4 delta ORF1/2 infection group was significantly higher than that seen in either the control group or the AB4 WT-infection group on days 1 and 2 pi (*p *< 0.001). In addition, the decrease in IL-8 mRNA levels seen in the Ab4 WT infection group was attenuated in the Ab4 ΔORF1/2 infection group, and Tbet levels in the Ab4 ΔORF1/2 infection group were higher compared to both the Ab4 WT infection group and the controls during this period although not quite reaching significance (*p*-values: 0.08 on day 3 and 0.07 on day 5).

In nasal secretions, IL-4 was not detected in any ponies while IL-10 and IFN-α could be detected in individual ponies of both infection groups on day 2 pi (Additional file 2: Table S1). Overall levels for both IL-10 and IFN-α were not significantly different from controls because increases were only observed in individual animals of each group. IFN-α was found in 5 out of 7 animals in the Ab4 WT-infected group and 4 out of 7 animals in the Ab4 ΔORF1/2-infected group, while IL-10 was detectable at relatively low levels in 3 out of 7 animals in the Ab4 WT-infected group and 2 out of 7 animals in the Ab4 ΔORF1/2-infected group (Additional file 1: Table S1). IFN-α and IL-10 were not detected on any other day in any animal.

### Non-specific and EHV-1-specific lymphoproliferative responses

**Figure 5:**
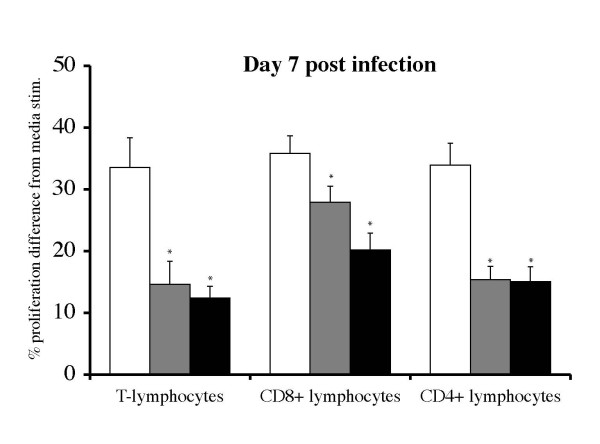
**Lymphoproliferative responses to in vitro stimulation with PHA on day 7 post infection**. Controls (*n *= 5): white bars, Ab4 WT infected (*n *= 7): grey bars, Ab4 delta ORF1/2 infected (*n *= 7): black bars. Data are displayed as means + SEM. A statistical difference from the control group is indicated by * (*p *< 0.05).

Infection of ponies with either virus suppressed responses in the two infection groups to PHA on day 7 pi when compared to PBMCs of controls (Figure 5). This immunosuppressive effect disappeared by day 21 pi. Decreased proliferative responses were observed only in T-lymphocytes for both infection groups when compared to controls ( *p *< 0.0001), while B-lymphocytes were not affected (data not shown). Within the T-lymphocyte population, both CD4+ and CD8+ lymphocytes were affected (Figure 5) and the immunosuppression occurred at a PHA concentration of both 1 μg/mL and 0.2 μg/mL (data not shown). Memory responses to EHV-1 that were above levels seen in control animals were observed in T-lymphocytes of both infection groups by day 96 pi, however the two infection groups were not significantly different (data not shown).

### Cytokine mRNA responses to in vitro re-stimulation with EHV-1

Re-stimulation of PBMCs collected on days 74 and 96 pi with WT Ab4 resulted in an increased IFN-γ and Granzyme B mRNA expression in both infection groups when compared to uninfected controls, although these differences did not reach significance (data not shown). No differences were seen for Perforin mRNA expression in PBMCs collected from the three experimental groups. Stimulation with PHA resulted in increases in cytokine mRNA expression for IFN-γ, Perforin and Granzyme B in all three groups when compared to media stimulation only, but no differences were observed between experimental groups (data not shown).

## Discussion

This study describes how infection of ponies with EHV-1 leads to viral modulation of equine immune responses and identifies two new EHV-1 genes with function in immunomodulation and disease attenuation. The sequential characterization of cytokine responses during the first seven days following infection with Ab4 WT virus shows that increases in pro-inflammatory (IL-1, IFN-α, TNF-α), T-helper 1 associated (IFN-γ,) and regulatory cytokines (TGF-β and IL-10) coincide with the biphasic increases in body temperature and the onset of respiratory disease and viremia respectively. In the period between, cytokine responses decreased in Ab4 WT-infected ponies, while decreases in IL-8 were attenuated in Ab4 ΔORF1/2 virus infected ponies and Tbet responses were increased compared to Ab4 WT-infected ponies and controls.

Similar to what has been reported in murine studies of EHV-1 infection [34], increases in cytokine/chemokine responses with inflammatory, antiviral (IL-1, TNF-α, (PBMCs) and IFN-α (nasal secretions)), and regulatory function (TGF-β (PBMCs) and IL-10 (nasal secretions)) were seen in the first 48 h following infection. With decreasing body temperatures, overall cytokine expression returned to baseline levels and cytokine mRNA, associated with cellular immune responses (IL-2 and Tbet) decreased markedly in Ab4 WT virus infected ponies. Additionally, a significant decrease of IL-8 mRNA was observed in AB4 WT infected ponies. Decreased levels of IL-8 mRNA expression are consistent with previous in vitro and murine reports that have attributed this phenomenon to the broad range of chemokine binding properties of the EHV-1 glycoprotein G [6]. Parallel with the onset viremia, increases in the mRNA expression of cytokines important for the development of CTL-responses (IFN-γ), as well as increases in the expression of regulatory cytokines (IL-10 and TGF-β) were observed in PBMCs of infected ponies. The latter is particularly interesting because we also found a non-specific immunosuppression of T-cell responses in both infection groups during this period. A study by Charan et al. [35] found that post-EHV-1 infection autologous sera collected from horses on days 3 to 21 pi contained increased levels of TGF-β and caused both non-specific and EHV-1 specific suppression of T-cell responses. This suppression was reversed by the addition of an antibody to TGF-β [35]. Non-specific T-cell suppression in both the Charan et al. study and our present study was temporary and disappeared by 21 pi. EHV-1 specific proliferative T-cell responses and EHV-1-specific IFN-γ and Granzyme B responses increased only marginally in infected ponies when compared with controls. Whether immunosuppressive effects of the virus caused these "weak" memory responses or whether our methodology was not adequate to enumerate EHV-1 specific memory responses remains to be determined. There are several studies in the literature that were able to detect significant CTL or IFN-γ responses post EHV-1 infection, however, responses were mostly significant in horses with prior exposure or vaccination for EHV-1 [2,28,36-39].

Antibody responses in all three experimental groups were measured by standard serum neutralizing test in addition to a kinetic ELISA that determined IgG subclass responses as previously described [18]. Antibody responses to EHV-1 infection in both infection groups increased significantly by day 14 pi. The IgGa and IgGb subclasses showed strong similarity to SN titer responses, with the IgGb subclass response most closely following the SN pattern consistent with prior observations [18,28]. Responses in the IgGc subclass, while significant compared to uninfected controls, were of very low amplitude and a role for this equine IgG subclass has not been defined yet. For IgG(T), it was notable that there was a pre-existing titer in all experimental groups despite the fact that SN titers were negligible at this time point, suggesting that IgG(T) responses do not contribute substantially to SN titers or to protective humoral immunity to EHV-1. As it has previously been hypothesized that the IgGb/IgG(T) ratio may predict protection from EHV-1 infection [17], IgGb/IgG(T) ratios were determined in our study, but were negligible, most likely due to the low IgG(T) titers, and hence, may not be of much value for assessing immune responses (data not shown).

Deletion of the ORF1/2 genes from EHV-1 strain Ab4 attenuated EHV-1 disease and modulated IL-8 and Tbet responses. Ab4 ΔORF1/2 infected ponies showed significantly shortened primary fevers, and reduced nasal viral shedding, despite the fact that both viruses show similar in vitro growth kinetics. The significant reduction in nasal viral shedding could be correlated with the increase in IgGa production in the Ab4 ΔORF1/2 infected ponies. However no significant increases in either SN titers or IgGb, which are typically associated with increased clearance of virus in nasal secretions, [40] were observed. Alternatively, deletion of the ORF1/2 genes resulted in increases of the chemokine IL-8 on days 3, 4 and 5 pi when compared to infection with the Ab4 WT-virus. This observation suggests that the ORF1/2 genes may function in preventing chemotaxis, as has been described for EHV-1 infection and so far been attributed to the gG envelope protein only [6]. Deletion of the ORF1/2 genes may thus have resulted in increased chemotaxis of phagocytic cells and thus more rapid removal of infectious virus, reflected by decreased viral shedding. Clearly, the direct effects of these genes at the respiratory epithelium need to be further investigated.

In addition, Ab4 ΔORF1/2 infected ponies exhibited significantly increased Tbet mRNA expression in the Ab4 WT-infected group. We speculate that the deletion of the ORF1/2 genes may have allowed for the development of TH 1 polarized immune responses, although no other data were generated in the present study to support this. IFN-γ responses to EHV-1 re-stimulation were not found to be significantly different between infection groups, suggesting that ORF1/2 deletion does not lead to the induction of a stronger memory CTL response. However, IFN-γ mRNA expression does not correlate perfectly with memory CTL responses [41] and standard chromium release assays were not performed in this study. Finally, while SN antibody responses and most IgG isotype responses following infection were similar for both infection groups, serum IgGa responses were significantly increased in the Ab4 ΔORF1/2 group on days 14 and 21 pi, which might again indicate that the deletion of the ORF1/2 genes promoted a stronger development of IgG isotype responses associated with the development of TH-1 type immune responses [3].

In conclusion, we found that infection of ponies with EHV-1 leads to modulation of equine immune responses, which are closely correlated with disease pathogenesis. In addition we have identified the ORF1/2 genes as two additional genes which function in attenuating selected clinical disease and nasal viral shedding and modulating IL-8 and Tbet responses. It is clear that EHV-1, like other α-herpesviruses has employed a plethora of mechanisms to evade the host immune system, and studying the in vivo function of responsible gene products is the first step for generation of a new class of modified live vaccines for protection from EHV-1.

## Competing interests

The authors declare that they have no competing interests.

## Authors' contributions

GSH and DPL provided the funding for the study. GSH prepared the manuscript, performed the proliferative and re-stimulation experiments and supervised the project. SBH performed the animal experiment and all clinical sampling. BW performed the antibody and cytokine Luminex analysis. DWH performed the cytokine real-time analysis. GvdW and NO generated and tested the deletion mutant virus, and GvdW provided the corresponding figures for the manuscript. LSG helped with the sampling of the different experimental groups. SR provided the statistical analysis. All authors read and approved the final manuscript.

## Supplementary Material

Additional file 1 **In vitro growth characteristics of the Ab4 WT and ΔORF1/2 viruses**. Titers were measured on RK-13 cells and represent results of 3 repeats. Ab4 WT virusare represented as squares, Ab4 delta ORF1/2 are represented as diamonds. Intracellular virus titers (a) and extracellular viral titers (b) are depicted. Data are displayed as means ± STDEV. Click here for file

Additional file 2 **Table S1**. IFN-α, IL-10 and IL-4 levels in nasal secretions collected on day 2 pi. Click here for file
