# Supplementary material for: Evaluation of immune responses following infection of ponies with an EHV-1 ORF1/2 deletion mutant
Source: Vet Res. 2011 Feb 7;42(1):23. doi: 10.1186/1297-9716-42-23 (PMC3045331; doi:10.1186/1297-9716-42-23)

Additional file 1: Figure S1. In vitro growth characteristics of the Ab4 WT and ΔORF1/2 viruses.
Titers were measured on RK-13 cells and represent results of 3 repeats. Ab4 WT virusare represented as squares, Ab4 delta ORF1/2 are represented as diamonds . Intracellular virus titers (a) and extracellular viral titers (b) are depicted. Data are displayed as means ± STDEV.

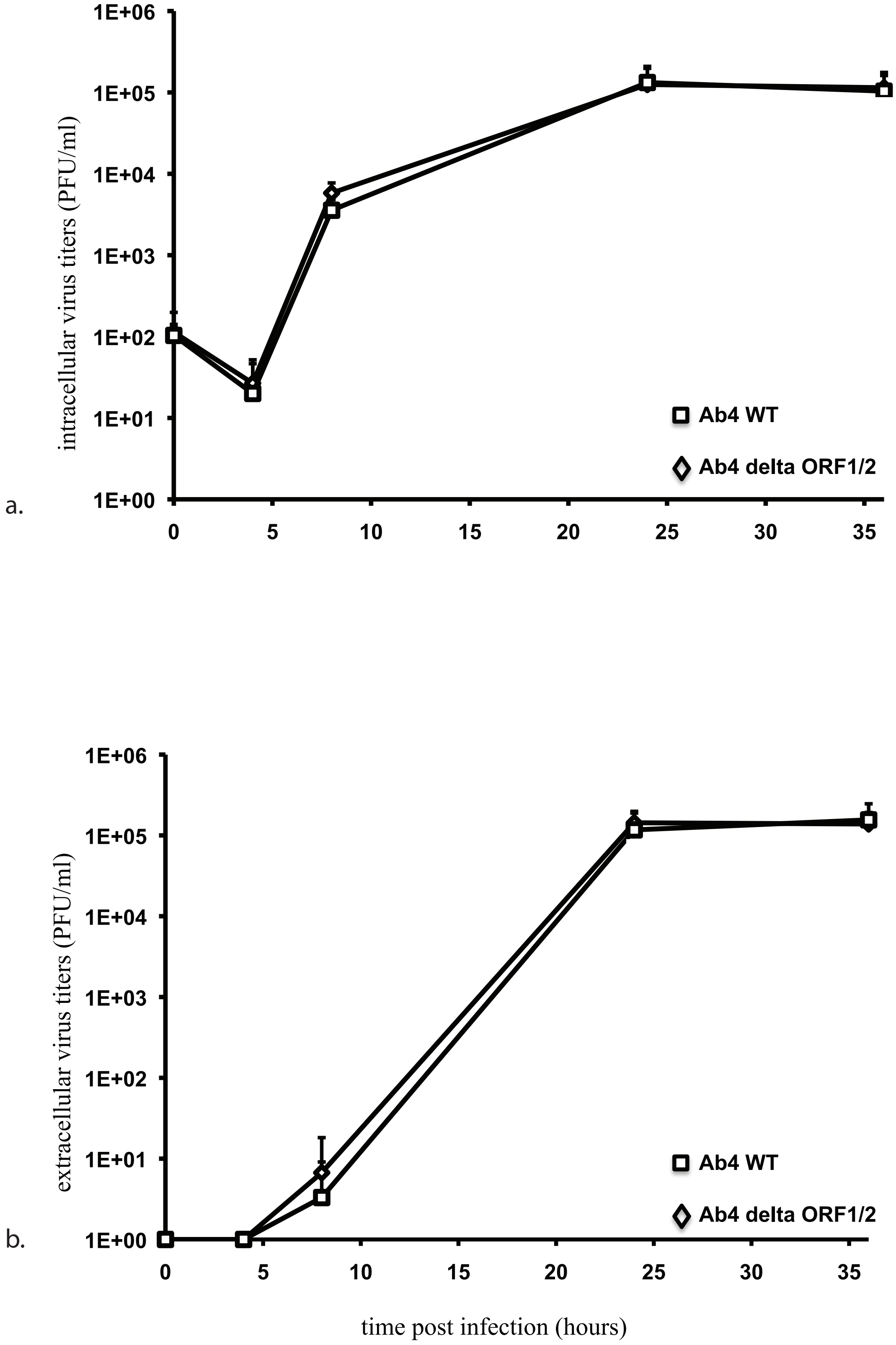

Supplement: Additional file 1 — In vitro growth characteristics of the Ab4 WT and ΔORF1/2 viruses. Titers were measured on RK-13 cells and represent results of 3 repeats. Ab4 WT virusare represented as squares, Ab4 delta ORF1/2 are represented as diamonds. Intracellular virus titers (a) and extracellular viral titers (b) are depicted. Data are displayed as means ± STDEV. [file 1297-9716-42-23-S1.PDF]
