# Supplementary material for: Evaluation of immune responses following infection of ponies with an EHV-1 ORF1/2 deletion mutant
Source: Vet Res. 2011 Feb 7;42(1):23. doi: 10.1186/1297-9716-42-23 (PMC3045331; doi:10.1186/1297-9716-42-23)
Supplement: Additional file 2 — Table S1. IFN-α, IL-10 and IL-4 levels in nasal secretions collected on day 2 pi. [file 1297-9716-42-23-S2.DOC]

**Additional file 2: Table S1**

**IFN-α, IL-10 and IL-4 levels in nasal secretions collected on day 2 pi.**

| **Group** | **Pony #** | **IFN-α (pg/mL)** | **IL-10 (pg/mL)** | **IL-4 (pg/mL)** |
| --- | --- | --- | --- | --- |
| **Controls** | 151 | <13 | <46 | <117 |
|  | 152 | <13 | <46 | <117 |
|  | 153 | <13 | <46 | <117 |
|  | 154 | <13 | <46 | <117 |
|  | 158 | <13 | <46 | <117 |
| **Ab4 Wt** | 132 | 1101 | 109 | <117 |
|  | 133 | <13 | 361 | <117 |
|  | 139 | 116 | 47 | <117 |
|  | 141 | 9981 | <46 | <117 |
|  | 142 | 106 | <46 | <117 |
|  | 144 | 1439 | <46 | <117 |
|  | 150 | <13 | <46 | <117 |
| **Ab4 delta ORF1/2** | 134 | <13 | <46 | <117 |
|  | 137 | 5912 | 80 | <117 |
|  | 138 | 3433 | 23 | <117 |
|  | 147 | 2469 | <46 | <117 |
|  | 156 | <13 | <46 | <117 |
|  | 157 | 304 | <46 | <117 |
|  | 159 | <13 | <46 | <117 |
